# *trans*-Acetyl­dicarbon­yl(η^5^-cyclo­penta­dien­yl)(methyl­diphenyl­phosphane)molybdenum(II)

**DOI:** 10.1107/S1600536812034307

**Published:** 2012-08-08

**Authors:** Matthew T. Whited, Joseph W. Boerma, Michael J. McClellan, Christian E. Padilla, Daron E. Janzen

**Affiliations:** a Department of Chemistry, Carleton College, 1 N. College Street, Northfield, MN 55057, USA; b Department of Chemistry, St Catherine University, 2004 Randolph Avenue, St. Paul, MN 55105, USA

## Abstract

The title compound, [Mo(C_5_H_5_)(C_2_H_3_O)(C_13_H_13_P)(CO)_2_], was prepared by reaction of [Mo(CH_3_)(C_5_H_5_)(CO)_3_] with methyl­diphenyl­phosphane. The Mo^II^ atom exhibits a four-legged piano-stool coordination geometry with the acetyl and phosphane ligands *trans* to each other. There are several inter­molecular C—H⋯O hydrogen-bonding inter­actions involving carbonyl and acetyl O atoms as acceptors. A close nearly parallel π–π inter­action between the cyclo­penta­dienyl plane and the phenyl ring of the phosphane ligand is present, with an angle of 6.4 (1)° between the two least-squares planes. The centroid-to-centroid distance between these groups is 3.772 (3) Å, and the closest distance between two atoms of these groups is 3.449 (4) Å. Since each Mo complex is engaged in two of these inter­actions, the complexes form an infinite π-stack coincident with the *a* axis.

## Related literature

The synthesis of the title compound has been reported previously and its reactivity studied, though no structural information was provided (Adams *et al.*, 1997 ▶; Barnett *et al.*, 1972 ▶). A related structure has been reported for the triphenyl­phosphane-substituted version of the title compound (Churchill & Fennessey, 1968 ▶). For synthetic details, see: Gladysz *et al.* (1979 ▶).
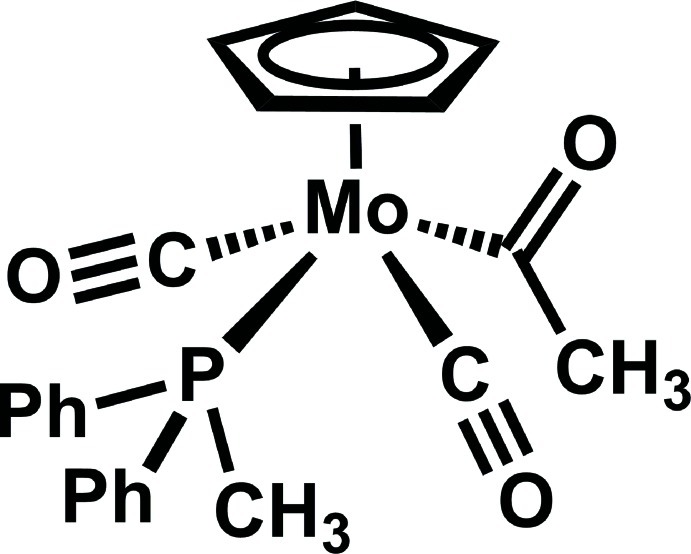


## Experimental

### 

#### Crystal data

[Mo(C_5_H_5_)(C_2_H_3_O)(C_13_H_13_P)(CO)_2_]
*M* *_r_* = 460.32
Orthorhombic, 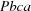

*a* = 11.482 (7) Å
*b* = 17.648 (10) Å
*c* = 20.771 (12) Å
*V* = 4209 (4) Å^3^
*Z* = 8
Mo *K*α radiation
μ = 0.72 mm^−1^
*T* = 193 K
0.44 × 0.24 × 0.24 mm

#### Data collection

Rigaku XtaLAB mini diffractometer
Absorption correction: multi-scan (*REQAB*; Rigaku, 1998 ▶) *T*_min_ = 0.687, *T*_max_ = 0.842
40944 measured reflections
4809 independent reflections
4311 reflections with *F* ^2^ > 2σ(*F* ^2^)
*R*_int_ = 0.037

#### Refinement

*R*[*F* ^2^ > 2σ(*F* ^2^)] = 0.032
*wR*(*F* ^2^) = 0.072
*S* = 1.13
4809 reflections
246 parameters
H-atom parameters constrained
Δρ_max_ = 0.63 e Å^−3^
Δρ_min_ = −0.67 e Å^−3^

### 

Data collection: *CrystalClear* (Rigaku Americas and Rigaku, 2011 ▶); cell refinement: *CrystalClear*; data reduction: *CrystalClear*; program(s) used to solve structure: *SIR2004* (Burla *et al.*, 2005 ▶); program(s) used to refine structure: *SHELXL97* (Sheldrick, 2008 ▶); molecular graphics: *CrystalStructure* (Rigaku Americas and Rigaku, 2010 ▶); software used to prepare material for publication: *CrystalStructure*.

## Supplementary Material

Crystal structure: contains datablock(s) global, I. DOI: 10.1107/S1600536812034307/wm2666sup1.cif

Supplementary material file. DOI: 10.1107/S1600536812034307/wm2666Isup2.cdx

Structure factors: contains datablock(s) I. DOI: 10.1107/S1600536812034307/wm2666Isup3.hkl

Additional supplementary materials:  crystallographic information; 3D view; checkCIF report

## Figures and Tables

**Table 1:** Selected bond lengths (Å)

Mo1—P1	2.4619 (15)
Mo1—C1	2.327 (3)
Mo1—C2	2.352 (3)
Mo1—C3	2.399 (3)
Mo1—C4	2.375 (3)
Mo1—C5	2.348 (3)
Mo1—C6	2.252 (3)
Mo1—C8	1.974 (3)
Mo1—C9	1.966 (3)
O1—C6	1.216 (3)
O2—C8	1.153 (4)
O3—C9	1.160 (3)

**Table 2:** Hydrogen-bond geometry (Å, °)

*D*—H⋯*A*	*D*—H	H⋯*A*	*D*⋯*A*	*D*—H⋯*A*
C10—H10*A*⋯O1^i^	0.98	2.41	3.346 (3)	159
C16—H16⋯O2^ii^	0.95	2.42	3.256 (3)	147
C3—H3⋯O1^iii^	1.00	2.45	3.390 (4)	156

Symmetry codes: (i) 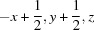; (ii) 
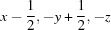
; (iii) 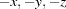.

**Fig. 1.**
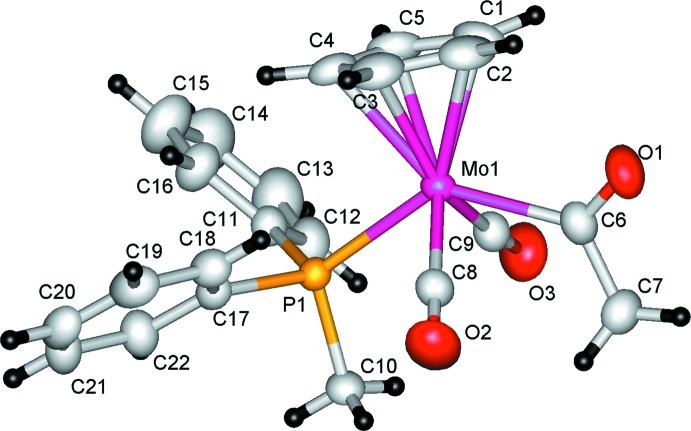
Numbered thermal ellipsoid plot (50% probability ellipsoids for non-H atoms) of the structure of (I).

**Fig. 2.**
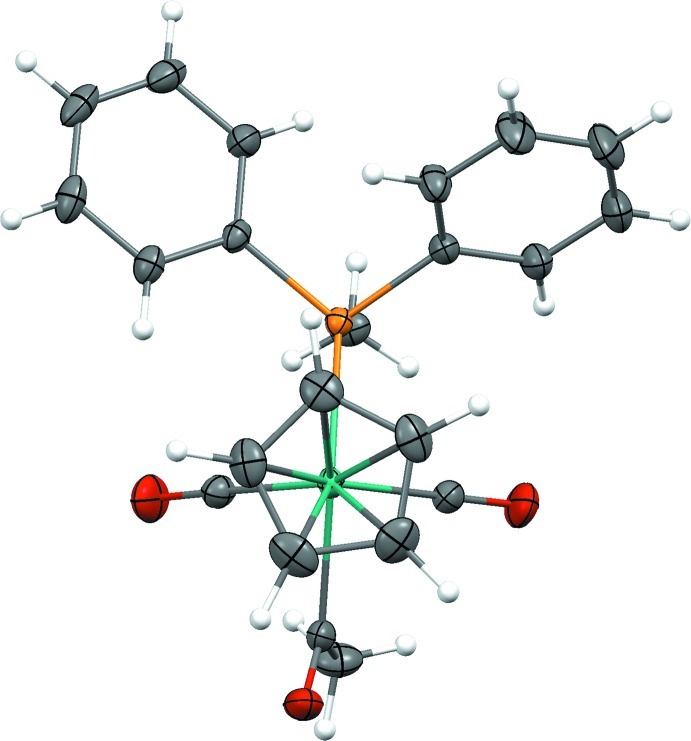
View of (I) perpendicular to Cp least-squares plane showing trans CO orientation.

**Fig. 3.**
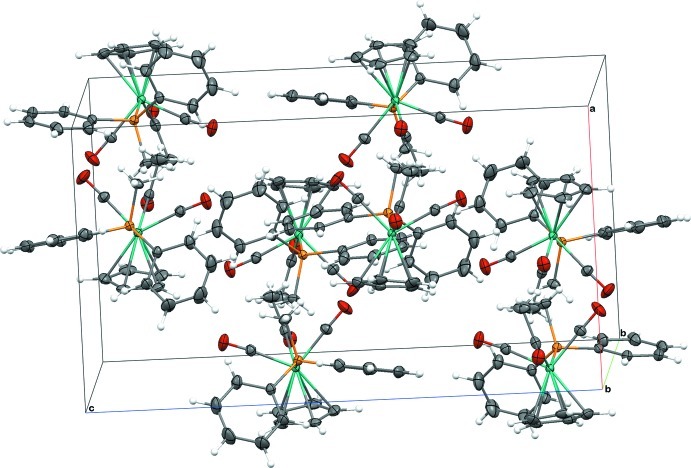
Unit cell packing diagram of (I) (50% probability ellipsoids).

**Fig. 4.**
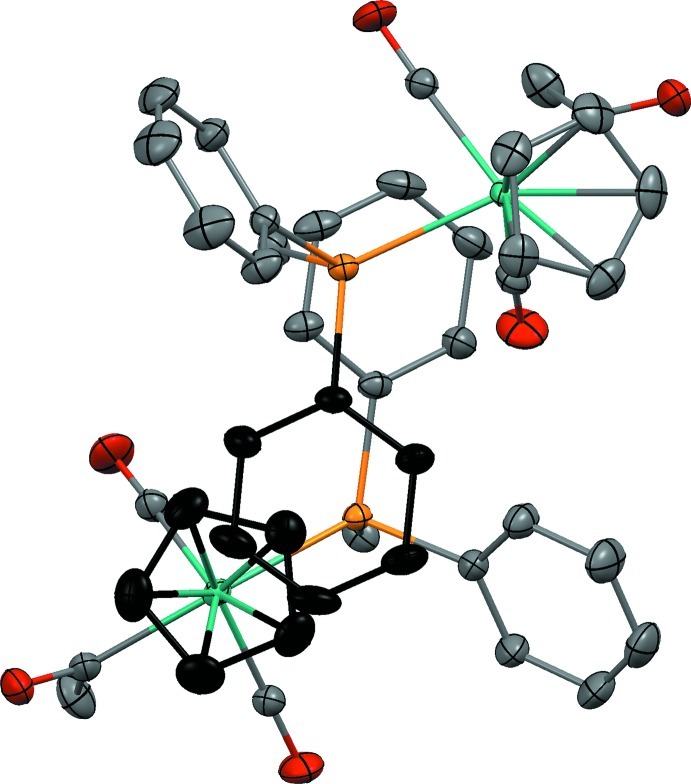
View of two complexes of (I) showing the π overlap (black atoms) of the phenyl ring of one molecule and the Cp ring of another molecule (symm. codes x, y, z for Cp, -1/2 + x, 1/2 - y, -z for Ph ring). H atoms omitted for clarity.

**Fig. 5.**
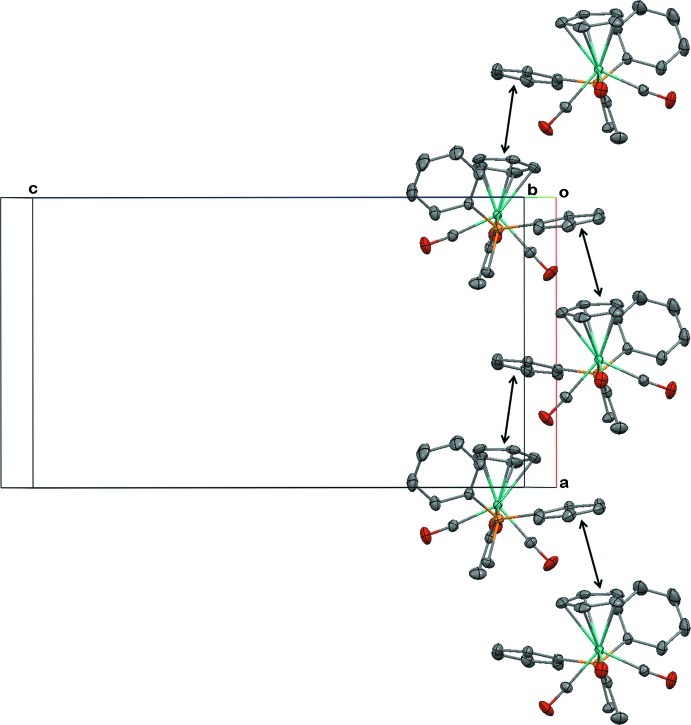
View of infinite intermolecular π–π stacking in (I) between Cp and Ph groups of neighboring complexes along the a-axis.

## supplementary crystallographic information

### Comment

Synthesis of the title complex, [Mo(C_5_H_5_)(C_2_H_3_O)(CO)_2_(C_13_H_13_P)], (I), has been previously reported and its reactivity studied, though no structural information was provided (Adams *et al.*, 1997; Barnett *et al.*, 1972).

The molecular structure of (Fig. 1), consists of a Mo(II) atom coordinated to a cyclopentadienyl ring in an η^5^ fashion, two CO ligands, one PMePh_2_ ligand, and one acetyl ligand. The orientation of the CO ligands can be described as *trans*. A view of the *trans* CO ligand orientation is shown in Fig. 2. The Mo—Cp centroid distance is 2.02959 (17) Å. The methyl group of the acetyl group is oriented in a *syn* fashion relative to the orientation of the methyl group of the PMePh_2_ ligand. A unit cell packing diagram is shown in Fig. 3.

There are several particularly short intermolecular distances involving H atoms. One short contact (2.421 Å) is present between O2 of a carbonyl ligand and H16 of a phenyl group (symmetry code: *x* - 1/2, -*y* + 1/2, -*z*). Another short contact (2.414 Å) involves O1 of the acetyl group and H10A of the methyl group of a PMePh_2_ ligand (symmetry code: -*x* + 1/2, *y* + 1/2,*z*). A third short contact (2.453 Å) is present between O1 of the acetyl group and H3 of a Cp ring (symmetry code: -*x*, -*y*, -*z*).

A close nearly parallel π—π intermolecular interaction between the Cp plane and the phenyl ring of a PMePh_2_ ligand is present. The angle between the least-squares planes of these close π systems is 6.4 (1)° (Cp ring plane composed of atoms C1–C5; phenyl ring plane composed of atoms C17–C22). While the Cp centroid–phenyl ring centroid distance is 3.772 (3) Å, the closest distance between these groups is 3.449 (4) Å between C20 and C5 (symmetry code: 1/2 + *x*, 1/2 - *y*, -*z*). Each Mo complex is engaged in two of these interactions, forming an infinite stack that is coincident with the *a*-axis. Fig. 4 shows the π overlap of neighboring molecules. Fig. 5 shows the infinite π-stacking interaction.

A related structure has been reported for the triphenylphosphane-substituted version of (I) (Churchill & Fennessey, 1968).

### Experimental

CpMo(CO)_3_(CH_3_). This compound was prepared by a modification of the method used by Gladysz *et al.* (1979) for the synthesis of a related iron compound. In an inert atmosphere glove box, [CpMo(CO)_3_]_2_ (181 mg, 0.370 mmol) was dissolved in THF (10 ml). LiEt_3_BH (0.87 ml, 1*M* in THF) was added dropwise by syringe with stirring, causing the evolution of H_2_ and a color change from purple to yellow. The solution was stirred for 30 min, then CH_3_I (75 µl, 1.2 mmol) was slowly added to the solution by micropipette, and the resulting solution was stirred for 2 h, causing a green–yellow precipitate to form. The solvent was removed *in vacuo* and the residues were extracted into pentane (2 × 15 ml) and filtered through a 2 cm plug of Al_2_O_3_ on a 30 ml fritted funnel, leaving a clear pale-yellow liquid. The Al_2_O_3_ was washed with about 10 ml of pentane, and the solvent was removed *in vacuo* to afford a solid yellow product (87 mg, 45%). IR and NMR (^1^H and ^13^C) spectral analyses confirmed the formation of the desired product.

CpMo(CO)_2_(PMePh_2_)(COCH_3_), (I). In an inert-atmosphere glove box, CpMo(CO)_3_(CH_3_) (87.2 mg, 0.335 mmol) was dissolved in 10 ml acetonitrile. PMePh_2_ (93 µl, 0.50 mmol) was added with stirring, and the resulting solution was stirred for 48 h. Solvent was removed *in vacuo*, leaving an orange oil, which was dissolved in diethyl ether and dried *in vacuo* to a yellow powder. The powder was triturated with pentane (5 ml) and isolated by filtration to afford the desired product in pure form as a yellow powder (79 mg, 51%), as confirmed by IR and NMR (^1^H, ^13^C, and ^31^P) spectral analyses. Crystalline material was obtained as yellow prisms by chilling a concentrated solution of (I) in diethyl ether.

### Refinement

H atoms were treated in calculated positions and refined in the riding model approximation with distances of C—H = 0.95, 1.00 and 0.98 Å for the phenyl, cyclopentadienyl and methyl groups, respectively, and with *U_iso_*(H) = *kU_eq_*(C), *k* = 1.2 for phenyl and cyclopentadienyl groups and 1.5 for methyl groups. Methyl group H atoms were allowed to rotate in order to find the best rotameric conformation. The maximum and minimum electron densities in the final difference Fourier map are located 0.87 and 0.75 Å, respectively, from atom Mo1.

### Crystal data

**Table:**

[Mo(C_5_H_5_)(C_2_H_3_O)(C_13_H_13_P)(CO)_2_]	*F*(000) = 1872.00
*M**_r_* = 460.32	*D*_x_ = 1.453 Mg m^−^^3^
Orthorhombic, *P**b**c**a*	Mo *K*α radiation, λ = 0.71075 Å
Hall symbol: -P 2ac 2ab	Cell parameters from 1664 reflections
*a* = 11.482 (7) Å	θ = 5.8–27.4°
*b* = 17.648 (10) Å	µ = 0.72 mm^−^^1^
*c* = 20.771 (12) Å	*T* = 193 K
*V* = 4209 (4) Å^3^	Prism, yellow
*Z* = 8	0.44 × 0.24 × 0.24 mm

### Data collection

**Table:**

Rigaku XtaLAB mini diffractometer	4311 reflections with *F*^2^ > 2σ(*F*^2^)
Detector resolution: 6.849 pixels mm^-1^	*R*_int_ = 0.037
ω scans	θ_max_ = 27.5°
Absorption correction: multi-scan (REQAB; Rigaku, 1998)	*h* = −14→14
*T*_min_ = 0.687, *T*_max_ = 0.842	*k* = −22→22
40944 measured reflections	*l* = −26→26
4809 independent reflections	

### Refinement

**Table:**

Refinement on *F*^2^	Secondary atom site location: difference Fourier map
*R*[*F*^2^ > 2σ(*F*^2^)] = 0.032	Hydrogen site location: inferred from neighbouring sites
*wR*(*F*^2^) = 0.072	H-atom parameters constrained
*S* = 1.13	*w* = 1/[σ^2^(*F*_o_^2^) + (0.0289*P*)^2^ + 2.9563*P*] where *P* = (*F*_o_^2^ + 2*F*_c_^2^)/3
4809 reflections	(Δ/σ)_max_ < 0.001
246 parameters	Δρ_max_ = 0.63 e Å^−^^3^
0 restraints	Δρ_min_ = −0.67 e Å^−^^3^
Primary atom site location: structure-invariant direct methods	

### Special details

**Table:**

Refinement. Refinement was performed using all reflections. The weighted *R*-factor (*wR*) and goodness of fit (*S*) are based on *F*^2^. *R*-factor (gt) are based on *F*. The threshold expression of *F*^2^ > 2.0 σ(*F*^2^) is used only for calculating *R*-factor (gt).

### Fractional atomic coordinates and isotropic or equivalent isotropic displacement parameters (Å^2^)

**Table:**

	*x*	*y*	*z*	*U*_iso_*/*U*_eq_	
Mo1	0.060442 (15)	0.126963 (10)	0.104324 (8)	0.02232 (7)	
P1	0.10879 (5)	0.26230 (3)	0.09372 (2)	0.02307 (11)	
O1	0.13079 (18)	−0.03933 (9)	0.11872 (8)	0.0440 (5)	
O2	0.26219 (18)	0.11759 (12)	0.00363 (10)	0.0571 (6)	
O3	0.16580 (19)	0.16063 (12)	0.23997 (8)	0.0519 (5)	
C1	−0.1051 (3)	0.06501 (17)	0.14181 (14)	0.0472 (7)	
C2	−0.0856 (3)	0.03902 (16)	0.07757 (16)	0.0483 (7)	
C3	−0.1018 (3)	0.09998 (17)	0.03529 (13)	0.0435 (6)	
C4	−0.12974 (19)	0.16438 (16)	0.07247 (13)	0.0401 (6)	
C5	−0.1332 (2)	0.14285 (16)	0.13805 (13)	0.0433 (6)	
C6	0.1703 (2)	0.02414 (13)	0.12514 (10)	0.0318 (5)	
C7	0.2962 (3)	0.02999 (15)	0.14855 (15)	0.0490 (7)	
C8	0.1896 (2)	0.12249 (13)	0.04171 (11)	0.0325 (5)	
C9	0.1317 (2)	0.14872 (13)	0.18841 (10)	0.0308 (5)	
C10	0.2599 (2)	0.28503 (13)	0.11366 (11)	0.0335 (5)	
C11	0.0223 (2)	0.32333 (12)	0.14697 (10)	0.0287 (5)	
C12	0.0544 (3)	0.33488 (14)	0.21134 (12)	0.0393 (6)	
C13	−0.0172 (4)	0.37648 (16)	0.25232 (13)	0.0545 (8)	
C14	−0.1206 (3)	0.40696 (19)	0.22946 (14)	0.0626 (9)	
C15	−0.1530 (3)	0.39641 (18)	0.16607 (15)	0.0573 (8)	
C16	−0.0814 (3)	0.35472 (15)	0.12501 (12)	0.0403 (6)	
C17	0.09086 (18)	0.30855 (13)	0.01478 (10)	0.0278 (5)	
C18	0.06705 (19)	0.26664 (15)	−0.04063 (11)	0.0340 (5)	
C19	0.0595 (3)	0.30314 (18)	−0.10050 (11)	0.0430 (7)	
C20	0.0746 (3)	0.38056 (17)	−0.10492 (12)	0.0456 (7)	
C21	0.0974 (3)	0.42270 (16)	−0.05024 (13)	0.0442 (7)	
C22	0.1062 (3)	0.38683 (14)	0.00936 (12)	0.0379 (6)	
H1	−0.1100	0.0326	0.1812	0.0566*	
H2	−0.0722	−0.0148	0.0646	0.0580*	
H3	−0.0998	0.0974	−0.0128	0.0522*	
H4	−0.1517	0.2152	0.0551	0.0481*	
H5	−0.1600	0.1754	0.1746	0.0519*	
H7A	0.3302	−0.0208	0.1512	0.0588*	
H7B	0.2977	0.0537	0.1912	0.0588*	
H7C	0.3413	0.0608	0.1183	0.0588*	
H10A	0.2717	0.3399	0.1105	0.0402*	
H10B	0.3121	0.2591	0.0835	0.0402*	
H10C	0.2768	0.2682	0.1576	0.0402*	
H12	0.1253	0.3143	0.2271	0.0471*	
H13	0.0047	0.3840	0.2960	0.0654*	
H14	−0.1694	0.4352	0.2576	0.0751*	
H15	−0.2238	0.4174	0.1505	0.0688*	
H16	−0.1038	0.3477	0.0814	0.0483*	
H18	0.0560	0.2134	−0.0378	0.0408*	
H19	0.0439	0.2744	−0.1382	0.0516*	
H20	0.0693	0.4049	−0.1456	0.0547*	
H21	0.1071	0.4760	−0.0533	0.0530*	
H22	0.1229	0.4159	0.0467	0.0455*	

### Atomic displacement parameters (Å^2^)

**Table:**

	*U*^11^	*U*^22^	*U*^33^	*U*^12^	*U*^13^	*U*^23^
Mo1	0.02136 (10)	0.02484 (10)	0.02077 (10)	−0.00183 (6)	0.00309 (6)	−0.00097 (7)
P1	0.0228 (3)	0.0254 (3)	0.0211 (3)	0.0007 (2)	−0.00052 (19)	0.0007 (2)
O1	0.0625 (13)	0.0279 (9)	0.0418 (10)	0.0008 (8)	−0.0040 (9)	0.0023 (8)
O2	0.0538 (13)	0.0667 (14)	0.0508 (12)	0.0067 (10)	0.0315 (10)	0.0057 (10)
O3	0.0704 (14)	0.0585 (12)	0.0267 (9)	0.0002 (10)	−0.0137 (9)	0.0010 (9)
C1	0.0326 (14)	0.0555 (17)	0.0535 (16)	−0.0186 (12)	0.0053 (12)	0.0097 (13)
C2	0.0282 (13)	0.0434 (15)	0.073 (2)	−0.0121 (11)	−0.0024 (13)	−0.0167 (14)
C3	0.0286 (13)	0.0616 (17)	0.0403 (14)	−0.0073 (12)	−0.0052 (11)	−0.0111 (13)
C4	0.0175 (11)	0.0498 (15)	0.0529 (15)	−0.0005 (10)	−0.0043 (10)	−0.0022 (12)
C5	0.0222 (12)	0.0583 (17)	0.0494 (15)	−0.0056 (11)	0.0118 (11)	−0.0113 (13)
C6	0.0408 (13)	0.0289 (11)	0.0257 (11)	0.0031 (10)	0.0073 (9)	0.0023 (9)
C7	0.0369 (15)	0.0408 (14)	0.0693 (19)	0.0089 (11)	−0.0017 (13)	0.0096 (14)
C8	0.0320 (12)	0.0313 (12)	0.0342 (12)	0.0011 (9)	0.0052 (10)	0.0034 (10)
C9	0.0339 (12)	0.0301 (11)	0.0284 (11)	0.0012 (9)	0.0015 (10)	0.0026 (9)
C10	0.0264 (12)	0.0325 (12)	0.0415 (13)	−0.0055 (9)	−0.0056 (10)	0.0041 (10)
C11	0.0340 (12)	0.0265 (11)	0.0257 (11)	0.0019 (9)	0.0016 (9)	−0.0007 (9)
C12	0.0521 (16)	0.0354 (13)	0.0303 (12)	0.0054 (11)	−0.0040 (11)	−0.0045 (10)
C13	0.080 (3)	0.0530 (17)	0.0304 (13)	0.0117 (16)	0.0023 (14)	−0.0119 (12)
C14	0.077 (3)	0.064 (2)	0.0466 (17)	0.0237 (18)	0.0154 (16)	−0.0158 (15)
C15	0.0537 (18)	0.068 (2)	0.0500 (17)	0.0300 (15)	0.0059 (14)	−0.0058 (15)
C16	0.0398 (14)	0.0490 (15)	0.0321 (13)	0.0147 (11)	0.0003 (10)	−0.0046 (11)
C17	0.0244 (11)	0.0352 (12)	0.0238 (10)	0.0036 (9)	0.0034 (8)	0.0051 (9)
C18	0.0307 (12)	0.0444 (14)	0.0270 (11)	−0.0010 (10)	−0.0014 (9)	0.0036 (10)
C19	0.0343 (13)	0.0688 (19)	0.0259 (12)	−0.0019 (12)	−0.0027 (10)	0.0049 (12)
C20	0.0321 (14)	0.0682 (19)	0.0364 (14)	0.0097 (12)	0.0043 (10)	0.0256 (13)
C21	0.0418 (15)	0.0444 (15)	0.0462 (15)	0.0108 (12)	0.0094 (12)	0.0185 (12)
C22	0.0455 (15)	0.0342 (13)	0.0340 (13)	0.0049 (11)	0.0047 (11)	0.0051 (10)

### Geometric parameters (Å, º)

**Table:**

Mo1—P1	2.4619 (15)	C17—C18	1.395 (4)
Mo1—C1	2.327 (3)	C17—C22	1.397 (4)
Mo1—C2	2.352 (3)	C18—C19	1.403 (4)
Mo1—C3	2.399 (3)	C19—C20	1.380 (5)
Mo1—C4	2.375 (3)	C20—C21	1.383 (4)
Mo1—C5	2.348 (3)	C21—C22	1.394 (4)
Mo1—C6	2.252 (3)	C1—H1	1.000
Mo1—C8	1.974 (3)	C2—H2	1.000
Mo1—C9	1.966 (3)	C3—H3	1.000
P1—C10	1.828 (3)	C4—H4	1.000
P1—C11	1.836 (3)	C5—H5	1.000
P1—C17	1.843 (3)	C7—H7A	0.980
O1—C6	1.216 (3)	C7—H7B	0.980
O2—C8	1.153 (4)	C7—H7C	0.980
O3—C9	1.160 (3)	C10—H10A	0.980
C1—C2	1.429 (5)	C10—H10B	0.980
C1—C5	1.413 (5)	C10—H10C	0.980
C2—C3	1.401 (5)	C12—H12	0.950
C3—C4	1.411 (4)	C13—H13	0.950
C4—C5	1.415 (4)	C14—H14	0.950
C6—C7	1.529 (4)	C15—H15	0.950
C11—C12	1.402 (4)	C16—H16	0.950
C11—C16	1.390 (4)	C18—H18	0.950
C12—C13	1.393 (4)	C19—H19	0.950
C13—C14	1.387 (5)	C20—H20	0.950
C14—C15	1.381 (5)	C21—H21	0.950
C15—C16	1.395 (4)	C22—H22	0.950
			
P1···O3	3.588 (3)	C2···H21^vii^	3.5742
O1···C1	3.310 (4)	C3···H2^i^	3.2486
O1···C2	2.969 (4)	C3···H10A^vii^	3.5224
O1···C8	3.342 (4)	C4···H10B^vii^	3.5725
O2···C6	3.194 (4)	C5···H20^vii^	3.5218
O2···C7	3.406 (4)	C6···H3^i^	3.2715
O2···C18	3.576 (4)	C6···H10A^ii^	3.3327
O3···C6	3.390 (4)	C6···H13^iii^	3.5838
O3···C7	3.341 (4)	C6···H14^iii^	2.8981
O3···C10	3.588 (4)	C6···H22^ii^	3.4551
O3···C12	3.383 (4)	C7···H10A^ii^	3.5336
C4···C16	3.575 (5)	C7···H14^iii^	2.9531
C7···C8	3.015 (4)	C7···H20^iv^	3.3399
C7···C9	2.940 (4)	C7···H22^ii^	3.0641
C8···C10	3.334 (4)	C8···H2^i^	3.2094
C8···C17	3.518 (4)	C8···H16^iv^	3.5279
C8···C18	3.373 (4)	C10···H3^iv^	3.3599
C9···C10	3.220 (4)	C10···H19^iv^	3.4630
C9···C11	3.437 (4)	C11···H7A^ix^	3.2309
C9···C12	3.436 (4)	C12···H7A^ix^	3.1301
C10···C12	3.234 (4)	C13···H1^x^	3.4102
C10···C22	3.322 (4)	C13···H7A^ix^	3.5078
C11···C14	2.794 (4)	C13···H10C^viii^	3.5690
C11···C22	3.218 (4)	C13···H19^vi^	3.5713
C12···C15	2.782 (5)	C14···H10C^viii^	3.5888
C13···C16	2.772 (4)	C14···H12^viii^	3.4638
C16···C17	3.133 (4)	C15···H21^xiii^	3.2914
C16···C22	3.276 (4)	C16···H21^xiii^	3.3506
C17···C20	2.798 (4)	C17···H4^iv^	3.3192
C18···C21	2.783 (5)	C18···H4^iv^	3.2589
C19···C22	2.771 (4)	C18···H10B^vii^	3.0935
O1···C3^i^	3.390 (4)	C19···H4^iv^	3.4631
O1···C10^ii^	3.346 (4)	C19···H5^iv^	3.5902
O1···C13^iii^	3.329 (4)	C19···H7C^vii^	3.4892
O1···C14^iii^	3.295 (4)	C19···H10B^vii^	3.0662
O2···C16^iv^	3.256 (4)	C20···H5^iv^	3.5154
O3···C5^v^	3.441 (4)	C20···H7C^vii^	2.8845
O3···C19^vi^	3.589 (4)	C21···H7C^vii^	3.2752
O3···C20^vi^	3.465 (4)	C22···H3^iv^	3.3875
C3···O1^i^	3.390 (4)	C22···H4^iv^	3.5719
C3···C21^vii^	3.491 (4)	C22···H7A^ix^	3.4448
C3···C22^vii^	3.486 (4)	C22···H21^xiii^	3.5620
C4···C20^vii^	3.551 (4)	H1···C13^iii^	3.4102
C4···C21^vii^	3.520 (4)	H1···H7B^viii^	2.8790
C4···C22^vii^	3.591 (4)	H1···H13^iii^	2.9263
C5···O3^viii^	3.441 (4)	H1···H14^xii^	3.4470
C5···C20^vii^	3.449 (4)	H1···H15^xii^	2.8593
C10···O1^ix^	3.346 (4)	H2···O2^i^	3.1709
C13···O1^x^	3.329 (4)	H2···C2^i^	3.4907
C14···O1^x^	3.295 (4)	H2···C3^i^	3.2486
C16···O2^vii^	3.256 (4)	H2···C8^i^	3.2094
C19···O3^xi^	3.589 (4)	H2···H2^i^	3.1975
C20···O3^xi^	3.465 (4)	H2···H3^i^	2.6799
C20···C4^iv^	3.551 (4)	H2···H13^iii^	3.4899
C20···C5^iv^	3.449 (4)	H2···H15^xii^	3.1778
C21···C3^iv^	3.491 (4)	H2···H18^i^	3.5528
C21···C4^iv^	3.520 (4)	H3···O1^i^	2.4532
C22···C3^iv^	3.486 (4)	H3···C2^i^	3.4843
C22···C4^iv^	3.591 (4)	H3···C6^i^	3.2715
Mo1···H7B	3.5141	H3···C10^vii^	3.3599
Mo1···H7C	3.4422	H3···C22^vii^	3.3875
Mo1···H18	3.3238	H3···H2^i^	2.6799
P1···H4	3.2066	H3···H10A^vii^	2.7424
P1···H12	2.9240	H3···H10B^vii^	3.0966
P1···H16	2.8797	H3···H22^vii^	3.2696
P1···H18	2.9289	H4···O2^vii^	3.3418
P1···H22	2.8864	H4···C17^vii^	3.3192
O1···H1	3.3073	H4···C18^vii^	3.2589
O1···H2	2.6237	H4···C19^vii^	3.4631
O1···H7A	2.4098	H4···C22^vii^	3.5719
O1···H7B	2.9389	H4···H10B^vii^	2.9426
O1···H7C	2.9947	H5···O3^viii^	2.6865
O2···H7C	2.7383	H5···C19^vii^	3.5902
O2···H10B	3.0530	H5···C20^vii^	3.5154
O2···H18	3.0344	H5···H7B^viii^	3.5515
O3···H7B	2.6231	H5···H19^vii^	3.5945
O3···H10C	2.8558	H5···H20^vii^	3.4692
O3···H12	2.7637	H7A···C11^ii^	3.2309
C1···H3	3.2623	H7A···C12^ii^	3.1301
C1···H4	3.2499	H7A···C13^ii^	3.5078
C2···H4	3.2350	H7A···C22^ii^	3.4448
C2···H5	3.2528	H7A···H10A^ii^	2.8504
C3···H1	3.2570	H7A···H12^ii^	3.3486
C3···H5	3.2543	H7A···H14^iii^	2.7577
C3···H18	3.0975	H7A···H20^iv^	3.4250
C4···H1	3.2500	H7A···H22^ii^	2.4989
C4···H2	3.2352	H7B···H1^v^	2.8790
C4···H16	3.2540	H7B···H5^v^	3.5515
C4···H18	3.2472	H7B···H14^iii^	2.7710
C5···H2	3.2502	H7B···H20^iv^	3.3394
C5···H3	3.2568	H7C···C19^iv^	3.4892
C6···H1	3.4260	H7C···C20^iv^	2.8845
C6···H2	3.1320	H7C···C21^iv^	3.2752
C8···H3	3.5380	H7C···H20^iv^	2.7456
C8···H7B	3.5574	H7C···H21^iv^	3.3995
C8···H7C	2.5979	H7C···H22^ii^	2.9857
C8···H10B	2.9238	H10A···O1^ix^	2.4134
C8···H18	2.7671	H10A···C3^iv^	3.5224
C9···H1	3.4534	H10A···C6^ix^	3.3327
C9···H5	3.3938	H10A···C7^ix^	3.5336
C9···H7B	2.5395	H10A···H3^iv^	2.7424
C9···H7C	3.2132	H10A···H7A^ix^	2.8504
C9···H10B	3.5830	H10A···H13^v^	3.3963
C9···H10C	2.7631	H10A···H14^v^	3.2858
C9···H12	3.0307	H10B···C4^iv^	3.5725
C10···H12	2.8641	H10B···C18^iv^	3.0935
C10···H22	3.1218	H10B···C19^iv^	3.0662
C11···H4	3.3578	H10B···H3^iv^	3.0966
C11···H5	3.3952	H10B···H4^iv^	2.9426
C11···H10A	2.9772	H10B···H18^iv^	2.9960
C11···H10C	3.0888	H10B···H19^iv^	2.9537
C11···H13	3.2807	H10C···C13^v^	3.5690
C11···H15	3.2782	H10C···C14^v^	3.5888
C11···H22	2.8885	H10C···H13^v^	3.4564
C12···H10A	3.2587	H10C···H14^v^	3.4887
C12···H10C	3.0251	H10C···H19^iv^	3.1828
C12···H14	3.2652	H12···C14^v^	3.4638
C12···H16	3.2612	H12···H7A^ix^	3.3486
C13···H15	3.2590	H12···H14^v^	3.1961
C14···H12	3.2645	H12···H15^v^	3.5750
C14···H16	3.2543	H12···H19^vi^	3.3397
C15···H13	3.2571	H13···O1^x^	2.7191
C16···H4	2.9705	H13···C6^x^	3.5838
C16···H5	3.4489	H13···H1^x^	2.9263
C16···H12	3.2617	H13···H2^x^	3.4899
C16···H14	3.2587	H13···H10A^viii^	3.3963
C16···H22	3.0519	H13···H10C^viii^	3.4564
C17···H4	3.3421	H13···H15^v^	3.3619
C17···H10A	2.9281	H13···H19^vi^	3.1440
C17···H10B	3.0416	H14···O1^x^	2.6463
C17···H16	2.7176	H14···C6^x^	2.8981
C17···H19	3.2788	H14···C7^x^	2.9531
C17···H21	3.2819	H14···H1^xiv^	3.4470
C18···H3	3.5950	H14···H7A^x^	2.7577
C18···H4	3.3290	H14···H7B^x^	2.7710
C18···H16	3.5094	H14···H10A^viii^	3.2858
C18···H20	3.2726	H14···H10C^viii^	3.4887
C18···H22	3.2626	H14···H12^viii^	3.1961
C19···H21	3.2515	H15···O2^vii^	3.2645
C20···H18	3.2696	H15···C1^xiv^	3.2673
C20···H22	3.2584	H15···C2^xiv^	3.4187
C21···H19	3.2503	H15···H1^xiv^	2.8593
C22···H10A	2.9516	H15···H2^xiv^	3.1778
C22···H16	2.9206	H15···H12^viii^	3.5750
C22···H18	3.2652	H15···H13^viii^	3.3619
C22···H20	3.2620	H15···H21^xiii^	3.0672
H1···H2	2.5988	H16···O2^vii^	2.4214
H1···H5	2.5882	H16···C8^vii^	3.5279
H2···H3	2.5706	H16···H21^xiii^	3.1651
H3···H4	2.5815	H18···H2^i^	3.5528
H3···H18	2.7676	H18···H10B^vii^	2.9960
H4···H5	2.5829	H19···O3^xi^	3.1109
H4···H16	2.4631	H19···C10^vii^	3.4630
H4···H18	3.0675	H19···C13^xi^	3.5713
H7C···H10B	3.5893	H19···H5^iv^	3.5945
H10A···H12	2.9819	H19···H10B^vii^	2.9537
H10A···H22	2.5452	H19···H10C^vii^	3.1828
H10C···H12	2.4015	H19···H12^xi^	3.3397
H12···H13	2.3407	H19···H13^xi^	3.1440
H13···H14	2.3348	H20···O3^xi^	2.8666
H14···H15	2.3309	H20···C5^iv^	3.5218
H15···H16	2.3400	H20···C7^vii^	3.3399
H16···H22	2.9569	H20···H5^iv^	3.4692
H18···H19	2.3505	H20···H7A^vii^	3.4250
H19···H20	2.3268	H20···H7B^vii^	3.3394
H20···H21	2.3324	H20···H7C^vii^	2.7456
H21···H22	2.3392	H21···O2^ix^	3.1448
O1···H3^i^	2.4532	H21···C2^iv^	3.5742
O1···H10A^ii^	2.4134	H21···C15^xiii^	3.2914
O1···H13^iii^	2.7191	H21···C16^xiii^	3.3506
O1···H14^iii^	2.6463	H21···C22^xiii^	3.5620
O1···H22^ii^	3.2953	H21···H7C^vii^	3.3995
O2···H2^i^	3.1709	H21···H15^xiii^	3.0672
O2···H4^iv^	3.3418	H21···H16^xiii^	3.1651
O2···H15^iv^	3.2645	H21···H21^xiii^	3.4149
O2···H16^iv^	2.4214	H21···H22^xiii^	3.2597
O2···H21^ii^	3.1448	H22···O1^ix^	3.2953
O3···H5^v^	2.6865	H22···C6^ix^	3.4551
O3···H19^vi^	3.1109	H22···C7^ix^	3.0641
O3···H20^vi^	2.8666	H22···H3^iv^	3.2696
C1···H15^xii^	3.2673	H22···H7A^ix^	2.4989
C2···H2^i^	3.4907	H22···H7C^ix^	2.9857
C2···H3^i^	3.4843	H22···H21^xiii^	3.2597
C2···H15^xii^	3.4187		
			
P1—Mo1—C1	132.06 (8)	P1—C11—C16	119.97 (17)
P1—Mo1—C2	141.25 (8)	C12—C11—C16	118.7 (3)
P1—Mo1—C3	108.32 (8)	C11—C12—C13	120.2 (3)
P1—Mo1—C4	84.99 (8)	C12—C13—C14	120.0 (3)
P1—Mo1—C5	97.15 (8)	C13—C14—C15	120.3 (3)
P1—Mo1—C6	132.27 (7)	C14—C15—C16	119.7 (3)
P1—Mo1—C8	79.07 (7)	C11—C16—C15	121.0 (3)
P1—Mo1—C9	78.24 (7)	P1—C17—C18	121.40 (19)
C1—Mo1—C2	35.55 (11)	P1—C17—C22	119.70 (17)
C1—Mo1—C3	58.15 (11)	C18—C17—C22	118.8 (2)
C1—Mo1—C4	58.19 (11)	C17—C18—C19	120.0 (3)
C1—Mo1—C5	35.19 (11)	C18—C19—C20	120.4 (3)
C1—Mo1—C6	90.84 (10)	C19—C20—C21	120.1 (3)
C1—Mo1—C8	144.62 (10)	C20—C21—C22	119.9 (3)
C1—Mo1—C9	97.72 (11)	C17—C22—C21	120.8 (3)
C2—Mo1—C3	34.29 (11)	Mo1—C1—H1	126.077
C2—Mo1—C4	57.45 (10)	C2—C1—H1	126.080
C2—Mo1—C5	58.26 (10)	C5—C1—H1	126.073
C2—Mo1—C6	85.01 (10)	Mo1—C2—H2	125.537
C2—Mo1—C8	110.71 (11)	C1—C2—H2	125.531
C2—Mo1—C9	129.45 (11)	C3—C2—H2	125.541
C3—Mo1—C4	34.38 (10)	Mo1—C3—H3	126.038
C3—Mo1—C5	57.76 (10)	C2—C3—H3	126.024
C3—Mo1—C6	112.95 (10)	C4—C3—H3	126.038
C3—Mo1—C8	100.46 (11)	Mo1—C4—H4	125.605
C3—Mo1—C9	153.22 (10)	C3—C4—H4	125.604
C4—Mo1—C5	34.85 (10)	C5—C4—H4	125.610
C4—Mo1—C6	142.42 (9)	Mo1—C5—H5	125.855
C4—Mo1—C8	121.21 (10)	C1—C5—H5	125.863
C4—Mo1—C9	125.13 (10)	C4—C5—H5	125.858
C5—Mo1—C6	124.71 (10)	C6—C7—H7A	109.468
C5—Mo1—C8	155.88 (10)	C6—C7—H7B	109.471
C5—Mo1—C9	96.06 (10)	C6—C7—H7C	109.470
C6—Mo1—C8	70.94 (9)	H7A—C7—H7B	109.475
C6—Mo1—C9	75.75 (9)	H7A—C7—H7C	109.467
C8—Mo1—C9	106.30 (11)	H7B—C7—H7C	109.475
Mo1—P1—C10	113.99 (8)	P1—C10—H10A	109.466
Mo1—P1—C11	113.15 (8)	P1—C10—H10B	109.470
Mo1—P1—C17	118.95 (8)	P1—C10—H10C	109.464
C10—P1—C11	104.38 (11)	H10A—C10—H10B	109.472
C10—P1—C17	102.14 (10)	H10A—C10—H10C	109.489
C11—P1—C17	102.46 (11)	H10B—C10—H10C	109.467
Mo1—C1—C2	73.17 (16)	C11—C12—H12	119.888
Mo1—C1—C5	73.24 (16)	C13—C12—H12	119.863
C2—C1—C5	107.2 (3)	C12—C13—H13	119.974
Mo1—C2—C1	71.28 (16)	C14—C13—H13	119.977
Mo1—C2—C3	74.69 (16)	C13—C14—H14	119.842
C1—C2—C3	108.6 (3)	C15—C14—H14	119.849
Mo1—C3—C2	71.02 (16)	C14—C15—H15	120.161
Mo1—C3—C4	71.91 (15)	C16—C15—H15	120.160
C2—C3—C4	107.8 (3)	C11—C16—H16	119.517
Mo1—C4—C3	73.71 (15)	C15—C16—H16	119.501
Mo1—C4—C5	71.53 (14)	C17—C18—H18	120.007
C3—C4—C5	108.5 (3)	C19—C18—H18	120.002
Mo1—C5—C1	71.57 (15)	C18—C19—H19	119.816
Mo1—C5—C4	73.62 (14)	C20—C19—H19	119.814
C1—C5—C4	107.9 (3)	C19—C20—H20	119.951
Mo1—C6—O1	120.81 (19)	C21—C20—H20	119.945
Mo1—C6—C7	122.44 (17)	C20—C21—H21	120.036
O1—C6—C7	116.7 (3)	C22—C21—H21	120.032
Mo1—C8—O2	177.0 (2)	C17—C22—H22	119.627
Mo1—C9—O3	175.0 (2)	C21—C22—H22	119.622
P1—C11—C12	121.15 (18)		
			
P1—Mo1—C1—C2	122.96 (9)	C3—Mo1—C5—C1	79.16 (13)
P1—Mo1—C1—C5	8.48 (17)	C3—Mo1—C5—C4	−36.69 (11)
C1—Mo1—P1—C10	141.40 (10)	C5—Mo1—C3—C2	−79.68 (13)
C1—Mo1—P1—C11	22.32 (10)	C5—Mo1—C3—C4	37.20 (11)
C1—Mo1—P1—C17	−97.99 (11)	C3—Mo1—C6—O1	−21.07 (19)
P1—Mo1—C2—C1	−95.59 (15)	C3—Mo1—C6—C7	159.70 (13)
P1—Mo1—C2—C3	20.6 (2)	C6—Mo1—C3—C2	37.97 (14)
C2—Mo1—P1—C10	−167.39 (13)	C6—Mo1—C3—C4	154.85 (10)
C2—Mo1—P1—C11	73.54 (14)	C8—Mo1—C3—C2	111.56 (12)
C2—Mo1—P1—C17	−46.77 (13)	C8—Mo1—C3—C4	−131.55 (12)
P1—Mo1—C3—C2	−166.59 (8)	C9—Mo1—C3—C2	−66.2 (3)
P1—Mo1—C3—C4	−49.71 (12)	C9—Mo1—C3—C4	50.6 (3)
C3—Mo1—P1—C10	−155.34 (7)	C4—Mo1—C5—C1	115.9 (2)
C3—Mo1—P1—C11	85.58 (9)	C4—Mo1—C5—C4	0.00 (12)
C3—Mo1—P1—C17	−34.73 (8)	C5—Mo1—C4—C3	−116.5 (2)
P1—Mo1—C4—C3	133.37 (11)	C5—Mo1—C4—C5	−0.00 (12)
P1—Mo1—C4—C5	−110.15 (10)	C4—Mo1—C6—O1	2.1 (3)
C4—Mo1—P1—C10	179.04 (7)	C4—Mo1—C6—C7	−177.14 (12)
C4—Mo1—P1—C11	59.96 (8)	C6—Mo1—C4—C3	−39.91 (19)
C4—Mo1—P1—C17	−60.34 (7)	C6—Mo1—C4—C5	76.57 (17)
P1—Mo1—C5—C1	−173.66 (9)	C8—Mo1—C4—C3	59.37 (15)
P1—Mo1—C5—C4	70.48 (11)	C8—Mo1—C4—C5	175.85 (10)
C5—Mo1—P1—C10	146.31 (8)	C9—Mo1—C4—C3	−154.79 (10)
C5—Mo1—P1—C11	27.24 (7)	C9—Mo1—C4—C5	−38.31 (16)
C5—Mo1—P1—C17	−93.07 (8)	C5—Mo1—C6—O1	44.6 (2)
P1—Mo1—C6—O1	−168.84 (11)	C5—Mo1—C6—C7	−134.60 (14)
P1—Mo1—C6—C7	11.92 (18)	C6—Mo1—C5—C1	−17.96 (16)
C6—Mo1—P1—C10	−6.49 (8)	C6—Mo1—C5—C4	−133.81 (10)
C6—Mo1—P1—C11	−125.57 (8)	C8—Mo1—C5—C1	107.1 (3)
C6—Mo1—P1—C17	114.13 (8)	C8—Mo1—C5—C4	−8.7 (3)
C8—Mo1—P1—C10	−57.82 (8)	C9—Mo1—C5—C1	−94.80 (12)
C8—Mo1—P1—C11	−176.90 (8)	C9—Mo1—C5—C4	149.35 (12)
C8—Mo1—P1—C17	62.79 (8)	C8—Mo1—C6—O1	−114.64 (18)
C9—Mo1—P1—C10	51.58 (7)	C8—Mo1—C6—C7	66.12 (15)
C9—Mo1—P1—C11	−67.50 (8)	C9—Mo1—C6—O1	132.15 (18)
C9—Mo1—P1—C17	172.19 (8)	C9—Mo1—C6—C7	−47.08 (14)
C1—Mo1—C2—C1	−0.00 (13)	Mo1—P1—C11—C12	83.56 (16)
C1—Mo1—C2—C3	116.2 (2)	Mo1—P1—C11—C16	−91.91 (15)
C2—Mo1—C1—C2	−0.00 (12)	Mo1—P1—C17—C18	−9.41 (18)
C2—Mo1—C1—C5	−114.5 (2)	Mo1—P1—C17—C22	173.40 (10)
C1—Mo1—C3—C2	−37.90 (11)	C10—P1—C11—C12	−40.92 (18)
C1—Mo1—C3—C4	78.99 (13)	C10—P1—C11—C16	143.61 (15)
C3—Mo1—C1—C2	36.53 (11)	C10—P1—C17—C18	117.05 (16)
C3—Mo1—C1—C5	−77.95 (13)	C10—P1—C17—C22	−60.14 (17)
C1—Mo1—C4—C3	−78.87 (14)	C11—P1—C17—C18	−135.02 (15)
C1—Mo1—C4—C5	37.61 (11)	C11—P1—C17—C22	47.78 (17)
C4—Mo1—C1—C2	77.24 (13)	C17—P1—C11—C12	−147.13 (15)
C4—Mo1—C1—C5	−37.24 (11)	C17—P1—C11—C16	37.40 (18)
C1—Mo1—C5—C1	0.00 (13)	Mo1—C1—C2—Mo1	−0.0
C1—Mo1—C5—C4	−115.9 (2)	Mo1—C1—C2—C3	−65.93 (16)
C5—Mo1—C1—C2	114.5 (2)	Mo1—C1—C5—Mo1	0.0
C5—Mo1—C1—C5	0.00 (12)	Mo1—C1—C5—C4	65.15 (14)
C1—Mo1—C6—O1	34.40 (17)	C2—C1—C5—Mo1	−65.80 (18)
C1—Mo1—C6—C7	−144.83 (15)	C2—C1—C5—C4	−0.6 (3)
C6—Mo1—C1—C2	−80.21 (12)	C5—C1—C2—Mo1	65.84 (19)
C6—Mo1—C1—C5	165.32 (12)	C5—C1—C2—C3	−0.1 (3)
C8—Mo1—C1—C2	−23.1 (3)	Mo1—C2—C3—Mo1	0.0
C8—Mo1—C1—C5	−137.59 (14)	Mo1—C2—C3—C4	−62.93 (15)
C9—Mo1—C1—C2	−155.94 (12)	C1—C2—C3—Mo1	63.71 (19)
C9—Mo1—C1—C5	89.59 (13)	C1—C2—C3—C4	0.8 (3)
C2—Mo1—C3—C2	0.00 (13)	Mo1—C3—C4—Mo1	−0.0
C2—Mo1—C3—C4	116.9 (2)	Mo1—C3—C4—C5	−63.53 (13)
C3—Mo1—C2—C1	−116.2 (2)	C2—C3—C4—Mo1	62.35 (18)
C3—Mo1—C2—C3	0.00 (12)	C2—C3—C4—C5	−1.2 (3)
C2—Mo1—C4—C3	−36.59 (12)	Mo1—C4—C5—Mo1	−0.0
C2—Mo1—C4—C5	79.89 (14)	Mo1—C4—C5—C1	−63.81 (14)
C4—Mo1—C2—C1	−79.49 (13)	C3—C4—C5—Mo1	64.94 (17)
C4—Mo1—C2—C3	36.69 (11)	C3—C4—C5—C1	1.1 (3)
C2—Mo1—C5—C1	38.48 (12)	P1—C11—C12—C13	−174.91 (15)
C2—Mo1—C5—C4	−77.37 (14)	P1—C11—C16—C15	175.00 (15)
C5—Mo1—C2—C1	−38.08 (11)	C12—C11—C16—C15	−0.6 (4)
C5—Mo1—C2—C3	78.10 (13)	C16—C11—C12—C13	0.6 (4)
C2—Mo1—C6—O1	−0.71 (17)	C11—C12—C13—C14	−0.3 (4)
C2—Mo1—C6—C7	−179.94 (16)	C12—C13—C14—C15	−0.1 (5)
C6—Mo1—C2—C1	98.48 (12)	C13—C14—C15—C16	0.2 (5)
C6—Mo1—C2—C3	−145.34 (13)	C14—C15—C16—C11	0.2 (5)
C8—Mo1—C2—C1	165.94 (10)	P1—C17—C18—C19	−176.91 (13)
C8—Mo1—C2—C3	−77.89 (14)	P1—C17—C22—C21	177.60 (15)
C9—Mo1—C2—C1	31.55 (17)	C18—C17—C22—C21	0.3 (4)
C9—Mo1—C2—C3	147.72 (12)	C22—C17—C18—C19	0.3 (3)
C3—Mo1—C4—C3	−0.00 (12)	C17—C18—C19—C20	−0.5 (4)
C3—Mo1—C4—C5	116.5 (2)	C18—C19—C20—C21	0.1 (4)
C4—Mo1—C3—C2	−116.9 (2)	C19—C20—C21—C22	0.6 (4)
C4—Mo1—C3—C4	−0.00 (12)	C20—C21—C22—C17	−0.8 (4)

Symmetry codes: (i) −*x*, −*y*, −*z*; (ii) −*x*+1/2, *y*−1/2, *z*; (iii) −*x*, *y*−1/2, −*z*+1/2; (iv) *x*+1/2, −*y*+1/2, −*z*; (v) *x*+1/2, *y*, −*z*+1/2; (vi) *x*, −*y*+1/2, *z*+1/2; (vii) *x*−1/2, −*y*+1/2, −*z*; (viii) *x*−1/2, *y*, −*z*+1/2; (ix) −*x*+1/2, *y*+1/2, *z*; (x) −*x*, *y*+1/2, −*z*+1/2; (xi) *x*, −*y*+1/2, *z*−1/2; (xii) −*x*−1/2, *y*−1/2, *z*; (xiii) −*x*, −*y*+1, −*z*; (xiv) −*x*−1/2, *y*+1/2, *z*.

### Hydrogen-bond geometry (Å, º)

**Table:**

*D*—H···*A*	*D*—H	H···*A*	*D*···*A*	*D*—H···*A*
C10—H10*A*···O1^ix^	0.98	2.41	3.346 (3)	159
C16—H16···O2^vii^	0.95	2.42	3.256 (3)	147
C3—H3···O1^i^	1.00	2.45	3.390 (4)	156

Symmetry codes: (i) −*x*, −*y*, −*z*; (vii) *x*−1/2, −*y*+1/2, −*z*; (ix) −*x*+1/2, *y*+1/2, *z*.
